# How genome complexity can explain the difficulty of aligning reads to genomes

**DOI:** 10.1186/1471-2105-16-S17-S3

**Published:** 2015-12-07

**Authors:** Vinhthuy Phan, Shanshan Gao, Quang Tran, Nam S Vo

**Affiliations:** 1 Department of Computer Science, University of Memphis, Memphis, TN 38152, USA

**Keywords:** short-read alignment, genome complexity, next-generation sequencing

## Abstract

**Background:**

Although it is frequently observed that aligning short reads to genomes becomes harder if they contain complex repeat patterns, there has not been much effort to quantify the relationship between complexity of genomes and difficulty of short-read alignment. Existing measures of sequence complexity seem unsuitable for the understanding and quantification of this relationship.

**Results:**

We investigated several measures of complexity and found that *length-sensitive measures *of complexity had the highest correlation to accuracy of alignment. In particular, the rate of distinct substrings of length *k*, where *k *is similar to the read length, correlated very highly to alignment performance in terms of precision and recall. We showed how to compute this measure efficiently in linear time, making it useful in practice to estimate quickly the difficulty of alignment for new genomes without having to align reads to them first. We showed how the length-sensitive measures could provide additional information for choosing aligners that would align consistently accurately on new genomes.

**Conclusions:**

We formally established a connection between genome complexity and the accuracy of short-read aligners. The relationship between genome complexity and alignment accuracy provides additional useful information for selecting suitable aligners for new genomes. Further, this work suggests that the complexity of genomes sometimes should be thought of in terms of specific computational problems, such as the alignment of short reads to genomes.

## Background

Advances in next-generation sequencing technologies have driven the development of computational approaches to address the problem of aligning short reads to reference genomes [1-10]. Even so, the alignment problem remains challenging due to the presence of genomic repeats that are much longer than reads. Yu et al. [11] evaluated alignment performance of several aligners on repetitive regions selected from CpG islands and concluded that long repeats seriously degraded alignment performance.

Researchers generally believe that the difficulty of aligning short reads is very much related to the complexity of genomes; it is easier to misalign short reads when the genomes of interest have long and complicated repeat patterns. While there has been an interest in measuring complexity of strings, recent attention has been focused on complexity of DNA sequences [12-15]. Whiteford et al. [15] utilized k-mer frequencies as a way to visualize and understand the complexity of genomes. Kurtz et al. [14], similarly, annotated plant genomes with *k*-mer frequencies so that repeat structures and characteristics can be easily visualized. With the same approach to understanding genome complexity, Chor et al. [13] analyzed k-mer spectra of over 100 species and observed multimodal spectra for regions with specific CG content characteristics. Unfortunately, these measures cannot be easily quantified and immediately adopted to study how complexity affects the difficulty of short-read alignment.

In a recent study, Becher et al. [12] introduced a measure known as the *I*-complexity, which seems most promising as a tool to correlate sequence complexity to difficulty of short-read alignment. The authors showed several interesting properties of this measure, including its closeness to the Lempel-Ziv complexity and its efficient computation in linear time. The *I*-complexity can be easily adopted for our purpose and will be used among others to understand how genome complexity affects the difficulty of short-read alignment.

In this paper, we propose measures of complexity that are best suited for the analysis and understanding of the difficulty of short-read alignment and how such measures might be helpful in selecting appropriate aligners for new genomes. The inspiration for this work lies in the observation that complex repeat structures in DNA that affect the performance of computational tasks are *length specific*. For instance, in finding regulatory motifs in DNA sequences, repeated structures of interest are around 8-25 characters long. On the other hand, in aligning reads to genomes, such repeats probably have little effect on the performance of aligners. This means that measures such as the *I*-complexity that are general and not length-specific might not be best.

## Methods

### *I*-complexity and *D*-complexity

Becher et al. [12] introduced the *I*-complexity as a measure of complexity of strings. It is proportional to the number of distinct substrings of the input string. Specifically, the *I*-complexity of a DNA sequence *g *is defined to be:

$$I\left(g\right)=\overset{\left|g\right|}{\underset{i=1}{\sum }}{\text{log}}_{4}\left(LCP\left[i\right]+2\right)-{\text{log}}_{4}\left(LCP\left[i\right]+1\right)$$

where *LCP *[*i*] is the length of the longest common prefix of the suffixes of *g *starting at positions *S*[*i*] and at *S*[*i *- 1], and *S *is the suffix array of *g*. The suffix array *S *of *g *stores implicitly lexicographically sorted suffixes of *g*; i.e. for *i < j, g*_*S*[*i*]*···|g|*_ (the suffix starting at index *S*[*i*]) is lexicographically smaller than *g*_*S*[*j*]*···|g|*_ (the suffix starting at index *S*[*j*]).

The somewhat non-intuitive definition of the *I*-complexity has some advantages. The authors established upper and lower bounds for *I*(*g*), and showed that it was close to the Lempel-Ziv complexity of *g*. Further, it can be computed in linear time because the suffix and *LCP *arrays can be constructed in linear time [16,17].

Although the *I*-complexity will be used in our attempt to explore the relationship between complexity and difficulty of alignment, we introduce a similar measure, *D*(*g*), counts directly the rate of distinct substrings:

$$D\left(g\right)=\frac{2\cdot |\left\{x\;:f\left(x\right)>0\right\}|}{|g|\cdot \left(|g|+1\right)}$$

where *f*(*x*) denotes the number of occurrences of *x *in *g*. To be precise, *D*(*g*) is equal to the total number of distinct substrings divided by the total number of substrings of *g. D*(*g*) can be computed in linear time, due to the following lemma.

**Lemma 1 **$|\left\{x:f\left(x\right)>0\right\}|=\sum_{i=1}^{\left|g\right|}i-\sum_{i=1}^{\left|g\right|}LCP\left[i\right]$

*Proof *Suppose that a substring *s *of *g *occurs exactly *k *times. Then, there will be a block of size *k *in the suffix array that corresponds to *k *suffixes that have *s *as the common prefix. More specifically, assume that *s *is the common prefix of the suffixes of *g *starting at positions S[i],S[i+1],⋯,S[i+k-1]. We will call the occurrence of *s *at position *S*[*i*] its *representative occurrence*, and its occurrences at S[i+1],S[i+2],⋯,S[i+k-1] its *repeat occurrences*.

Each *repeat occurrence *of *s *is a prefix of the longest common prefix of the suffixes starting at S[i+1],S[i+2],⋯,S[i+k-1]. This means, each *repeat occurrence *of *s *is accounted for uniquely by the values of LCP[i+1],LCP[i+2],⋯,LCP[i+k-1].

If we focus on a position, for example *i *+ 1, we can see that the longest common prefix between *S*[*i *+ 1] and *S*[*i*] (let's call it $p_{1\cdots j}$) accounts uniquely for *j repeat occurrences*, namely $p_{1},\;p_{1\cdots 2},\;\cdots \;,\;p_{1\cdots \left(j-1\right)}$. One of these is *s*; the rest are *repeat occurrences *of other substrings. Thus, each repeat occurrence is accounted for uniquely in some entry of *LCP *and each entry of *LCP *accounts uniquely for some repeat occurrences. That implies that $\sum_{i=1}^{\left|g\right|}LCP\left[i\right]$ accounts for the total repeat occurrences of all substrings of *g*.

Further, $\sum_{i=1}^{\left|g\right|}i$ is the total number of substrings of *g*, since there are exactly *i *substrings starting at position *i*. Thus, if we remove all repeat occurrences from the total number of substrings, we will get precisely the total number of *representative occurrences*. This means $|\left\{x:f\left(x\right)>0\right\}|=\sum_{i=1}^{\left|g\right|}-\sum_{i=1}^{\left|g\right|}LCP\left[i\right]$.

### Length-sensitive measures of complexity

In addition to the *I *and *D*, we introduce two notions of length-sensitive measures of genome complexity. The motivation is that, depending on which computational tasks that are affected by the complexity of genomes, only a narrow range of repeat lengths play an important role; for instance, one would expect repeats that affect the finding of regulatory motifs to be much shorter than those that affect the alignment reads to genomes. Given a number *k*, we define *D_k _*and *R_k _*as follows:

$$D_{k}\left(g\right)=\frac{\left|\left\{x\;:f\left(x\right)>0.\;|x|=k\right\}\right|}{|g|-k+1}$$

$$R_{k}\left(g\right)=\frac{\sum_{f\left(x\right)>1,\;|x|=k}f\left(x\right)}{|g|-k+1}$$

where *f *(*x*) is the number of occurrences of *x *in *g. D_k _*and *R_k _*measure the rates of distinct substrings and repeats, respectively, of length *k. R_k _*and *D_k _*are not exact "opposites" because *R_k _*does not account for non-repeats, whereas *D_k _*does. *R_k _*is related to the function *C*(*k, r*) proposed by Whiteford et al. [15]. *C*(*k, r*) is the count of *k*-mers repeating exactly *r *times. Therefore, $R_{k}=\sum_{r>1}r\cdot C\left(k,\;r\right)$. *D_k _*and *R_k _*can be computed in linear time and space using suffix and LCP arrays.

Lemma 2 |{x:f(x)>0,|x|=k}|=|{j:s[j]≤|g|-k+1andLCP[j]<k}|

*Proof *A *k*-substring of *g *must start at an index between 1 and *|g*|-*k *+1. Further, if *LCP *[*j*] <*k*, the *k*-prefix of the suffix starting at *S*[*j*] is different from the *k*-prefix of the suffix starting at *S*[*j *- 1]. Thus, each *j *for which *S*[*j*] ≤ |*g*| - *k *+ 1 and *LCP *[*j*] <*k *represents exactly one distinct *k*-substring.

On the other hand, if *S*[*j*] *> |g|-k*+1 or *LCP *[*j*] *≥ k*, then the *k*-substring starting at *S*[*j*] does not exist or is not distinct. Since LCP runs through all positions of *g*, all distinct *k*-substrings are uniquely accounted for.

**Lemma 3 **$\sum_{f\left(x\right)>1,\;|x|=k}f\left(x\right)=\sum_{|i,j|\in I_{k}}\left(j-i+2\right)$, *where I_k _is the set of intervals *[*i, j*]*'s, where i ≤ j, such that*

*1 LCP *[*u*] *≥ k for i ≤ u ≤ j*

*2 LCP *[*i - *1] <*k unless i *= 1

*3 LCP *[*j *+ 1] <*k unless j *= *|g|*

*Proof *A *k*-repeat is a substring *x *of length *k*, with *f *(*x*) *>*1. Since the suffix array *S *is sorted lexicographically, *S *forms consecutive runs of *k*-repeats, which are *k*-prefixes of the suffixes stored implicitly by *S*. More specifically, each interval [*i, j] *∈ *I_k _*corresponds to all occurrences of exactly one *k*-repeat. The number of occurrences for each *k*-repeat is exactly *j - i *+ 2.

*I_k _*can be computed in linear time by scanning the *LCP *array once. Note that the index of LCP runs from 1 to *|g|*, and *LCP *[1] = 0.

### Relating genome complexity to difficulty of aligning short reads to genomes

*I, D, D_k _*, and *R_k _*provide quantitative measures of complexity for each genome. Intuitively, the more distinct substrings a reference genome has (i.e. high values of *I, D*, and *D_k_*), the easier to align reads to the reference genome. Conversely, the more long repeats the genome has, the more difficult to align reads to it correctly (since the probability of mismatching of a read with a wrong substring is higher.)

We measure the performance of an alignment algorithm using *precision *and *recall*, where precision is defined as the fraction of aligned reads that are correct (i.e. number of correctly aligned reads divided by the total number of aligned reads); and recall is defined as the fraction of reads that are correctly aligned (i.e. number of correctly aligned reads divided by the total number of reads). These definitions were also used by Liu et al. [8].

To correlate complexity values to difficulty of alignment, for each measure of complexity, we computed the linear correlation between the complexity values of sequences in a diverse data set including 100 genomic sequences, and the actual performance for each of 10 popular aligners. A good measure of complexity will correlate highly with alignment performance.

## Results

### Aligners and genomic data

We selected from NCBI and EMBL-EBI databases a total of 100 genomic sequences from bacteria, plants, and eukaryotes (including human chromosomes) with diverse complexity. Detail information of these sequences is described in Tables 1, 2, and 3. "N" bases were removed from these genomic sequences because they were not real contents and constituted false long repeats that inappropriately affected the true complexity of the genomes.

**Table 1:** Information on the selected 100 genomic sequences [Part 1].

ID	Genome size	Description	Lineage	Source
AE017198	1992676	Lactobacillus johnsonii NCC 533,	Bacteria, Firmicutes	EBI
AJ270060	14497843	Arabidopsis thaliana DNA chr. 4, long arm	Eukaryota, Viridiplantae	EBI
AM055943	2013089	Toxoplasma gondii RH, genomic DNA chr. Ib	Eukaryota, Alveolata	EBI
AM263198	2814130	Listeria welshimeri serovar 6b str. SLCC5334	Bacteria, Firmicutes	EBI
AM269894	1347714	Eimeria tenella chr. 1, ordered contigs	Eukaryota, Alveolata	EBI
BA000004	4202352	Bacillus halodurans C-125 DNA,	Bacteria, Firmicutes	EBI
BN001302	4011161	TPA: Aspergillus nidulans FGSC A4 chr. II	Eukaryota, Fungi	EBI
BX284601	15072434	Caenorhabditis elegans Bristol N2 genomic chr., I	Eukaryota, Metazoa	EBI
CAID01000012	521582	Ostreococcus tauri WGS project CAID00000000 data, contig chr. 12	Eukaryota, Viridiplantae	EBI
CM000001	122678785	Canis lupus familiaris chr. 1	Eukaryota, Metazoa	EBI
CM000038	23914537	Canis lupus familiaris chr. 38	Eukaryota, Metazoa	EBI
CM000043	1786351	Cryptococcus neoformans var. neoformans B-3501A chr. 4	Eukaryota, Fungi	EBI
CM000071	19787792	Drosophila pseudoobscura pseudoobscura strain MV2-25 chr. 3	Eukaryota, Metazoa	EBI
CM000091	57791882	Rattus norvegicus strain BN/SsNHsdMCW chr. 20	Eukaryota, Metazoa	EBI
CM000110	11219875	Gallus gallus chr. 18	Eukaryota, Metazoa	EBI
CM000134	21712932	Oryza sativa (indica cultivar-group) chr. 9	Eukaryota, Viridiplantae	EBI
CM000152	6357299	Dictyostelium discoideum AX4 chr. 3	Eukaryota, Amoebozoa	EBI
CM000157	22324452	Drosophila yakuba strain Tai18E2 chr. 2L	Eukaryota, Metazoa	EBI
CM000158	21139217	Drosophila yakuba strain Tai18E2 chr. 2R	Eukaryota, Metazoa	EBI
CM000169	4918979	Aspergillus fumigatus Af293 chr. 1	Eukaryota, Fungi	EBI
CM000177	161428367	Bos taurus chr. 1	Eukaryota, Metazoa	EBI
CM000201	44081797	Bos taurus chr. 25	Eukaryota, Metazoa	EBI
CM000208	4054025	Trypanosoma brucei brucei strain 927/4 GUTat10.1 chr. 10	Eukaryota, Euglenozoa	EBI
CM000209	199526509	Mus musculus chr. 1	Eukaryota, Metazoa	EBI
CM000302	78773432	Macaca mulatta chr. 16	Eukaryota, Metazoa	EBI
CM000377	185838109	Equus caballus chr. 1	Eukaryota, Metazoa	EBI
CM000452	2067354	Plasmodium vivax chr. 11	Eukaryota, Alveolata	EBI
CM000515	118548696	Taeniopygia guttata chr. 1	Eukaryota, Metazoa	EBI
CM000530	16962381	Taeniopygia guttata chr. 13	Eukaryota, Metazoa	EBI
CM000572	46535552	Pongo abelii chr. 22	Eukaryota, Metazoa	EBI
CM000575	8914601	Fusarium graminearum PH-1 chr. 2	Eukaryota, Fungi	EBI
CM000580	4643527	Gibberella moniliformis 7600 chr. 3	Eukaryota, Fungi	EBI
CM000592	5212762	Fusarium oxysporum f. sp. Lycopersici 4287 chr. 4	Eukaryota, Fungi	EBI

**Table 2:** Information on the selected 100 genomic sequences [Part 2].

ID	Genome size	Description	Lineage	Source
CM000612	1002813	Phaeodactylum tricornutum CCAP 1055/1 chr. 9	Eukaryota, Stramenopiles	EBI
CM000638	3042585	Thalassiosira pseudonana CCMP1335 chr. 1	Eukaryota, Stramenopiles	EBI
CM000692	1385275	Saccharomyces kluyveri NRRL Y-12651 chr. F	Eukaryota, Fungi	EBI
CM000767	55460251	Sorghum bicolor chr. 8	Eukaryota, Viridiplantae	EBI
CM000769	60981646	Sorghum bicolor chr. 10	Eukaryota, Viridiplantae	EBI
CM000777	301354135	Zea mays chr. 1.	Eukaryota, Viridiplantae	EBI
CM000799	47997241	Oryctolagus cuniculus chr. 10	Eukaryota, Metazoa	EBI
CM000829	61220071	Sus scrofa chr. 18.	Eukaryota, Metazoa	EBI
CM000831	1255352	Drosophila virilis strain 15010-1051.88 chr. 6.	Eukaryota, Metazoa	EBI
CM000850	41906774	Glycine max chr. 17	Eukaryota, Viridiplantae	EBI
CM000875	44557958	Callithrix jacchus chr. 20	Eukaryota, Metazoa	EBI
CM000906	55886266	Ovis aries chr. 22	Eukaryota, Metazoa	EBI
CM000907	66770968	Ovis aries chr. 23	Eukaryota, Metazoa	EBI
CM000917	27037145	Nasonia vitripennis chr. 3	Eukaryota, Metazoa	EBI
CM001221	42630297	Medicago truncatula chr. 5.	Eukaryota, Viridiplantae	EBI
CM001222	23282162	Medicago truncatula chr. 6.	Eukaryota, Viridiplantae	EBI
CM001276	232296185	Macaca fascicularis chr. 1	Eukaryota, Metazoa	EBI
CM001294	65364038	Macaca fascicularis chr. 19	Eukaryota, Metazoa	EBI
CP000048	922307	Borrelia hermsii DAH,	Bacteria, Spirochaetes	EBI
CP000496	2740984	Scheffersomyces stipitis CBS 6054 chr. 2, complete sequence.	Eukaryota, Fungi	EBI
CP000828	6503724	Acaryochloris marina MBIC11017,	Bacteria, Cyanobacteria	EBI
CP001037	8234322	Nostoc punctiforme PCC 73102,	Bacteria, Cyanobacteria	EBI
CP001141	945026	Phaeodactylum tricornutum CCAP 1055/1 chr. 11, complete sequence.	Eukaryota, Stramenopiles	EBI
CP001681	5167383	Pedobacter heparinus DSM 2366,	Bacteria, Bacteroidetes	EBI
CP001699	9127347	Chitinophaga pinensis DSM 2588,	Bacteria, Bacteroidetes	EBI
CP001982	5097447	Bacillus megaterium DSM319,	Bacteria, Firmicutes	EBI
CP002287	7013095	Achromobacter xylosoxidans A8,	Bacteria, Proteobacteria	EBI
CP002987	4044777	Acetobacterium woodii DSM 1030,	Bacteria, Firmicutes	EBI
CP003170	9239851	Actinoplanes sp. SE50/110,	Bacteria, Actinobacteria	EBI
CP003348	4321753	Desulfitobacterium dehalogenans ATCC 51507,	Bacteria, Firmicutes	EBI
CP003872	5196935	Acidovorax sp. KKS102,	Bacteria, Proteobacteria	EBI
CR380954	1050361	Candida glabrata strain CBS138 chr. H complete sequence.	Eukaryota, Fungi	EBI
CU234118	7456587	Bradyrhizobium sp. ORS278,complete sequence.	Bacteria, Proteobacteria	EBI
CU329672	2452883	Schizosaccharomyces pombe chr. III, complete sequence	Eukaryota, Fungi	EBI

**Table 3:** Information on the selected 100 genomic sequences [Part 3].

ID	Genome size	Description	Lineage	Source
CU928173	1114666	Zygosaccharomyces rouxii strain CBS732 chr. A complete sequence.	Eukaryota, Fungi	EBI
DG000010	27390870	Oryzias latipes DNA, chr.10, strain: HdrR.	Eukaryota, Metazoa	EBI
FA000001	10049037	Drosophila melanogaster unordered unlocalized genomic scaffolds (chrUn)	Eukaryota, Metazoa	EBI
FM178379	3325165	Aliivibrio salmonicida LFI1238 chr. 1	Bacteria, Proteobacteria	EBI
FN543502	5346659	Citrobacter rodentium ICC168,	Bacteria, Proteobacteria	EBI
FN554974	4531609	Trypanosoma brucei gambiense DAL972 chr. 11, complete sequence	Eukaryota, Euglenozoa	EBI
FO082874	3568623	Babesia microti strain RI chr. III, complete sequence.	Eukaryota, Alveolata	EBI
FP929060	3108859	Clostridiales sp. SM4/1 draft genome.	Bacteria, Firmicutes	EBI
FR798980	512965	Leishmania braziliensis MHOM/BR/75/M2904, chr. 6	Eukaryota, Euglenozoa	EBI
GCA 000002035.2	60348388	Danio rerio genome assembly, chr1	Eukaryota, Metazoa	EBI
GCA 000151905.1	229507203	Gorilla gorGor3.1 chr. 1	Eukaryota	Ensembl
HE601630	9743550	Schistosoma mansoni strain Puerto Rico chr. 7,	Eukaryota, Metazoa	EBI
HE616744	1292049	Torulaspora delbrueckii CBS 1146 chr. 3,	Eukaryota, Fungi	EBI
HE616749	833973	Torulaspora delbrueckii CBS 1146 chr. 8,	Eukaryota, Fungi	EBI
HE806319	1449145	Tetrapisispora blattae CBS 6284 chr. 4,	Eukaryota, Fungi	EBI
HE978314	1290777	Kazachstania naganishii CBS 8797 chr. 1,	Eukaryota, Fungi	EBI
NC 003070.9	30427671	Arabidopsis thaliana chr. 1, complete sequence.	Eukaryota, Viridiplantae	NCBI
NC 007605	171823	Human herpesvirus 4 complete wild type genome.	Viruses, dsDNA viruses	NCBI
NC 008394.4	45064769	Oryza sativa Japonica Group DNA, chr. 1, complete sequence, cultivar: Nipponbare	Eukaryota, Viridiplantae	NCBI
NC 008397.2	30039014	Oryza sativa Japonica Group DNA, chr. 4, complete sequence, cultivar: Nipponbare	Eukaryota, Viridiplantae	NCBI
NC 008398.2	32124789	Oryza sativa Japonica Group DNA, chr. 5, complete sequence, cultivar: Nipponbare	Eukaryota, Viridiplantae	NCBI
NC 008399.2	30357780	Oryza sativa Japonica Group DNA, chr. 6, complete sequence, cultivar: Nippon bare	Eukaryota, Viridiplantae	NCBI
NC 008400.2	28530027	Oryza sativa Japonica Group DNA, chr. 7, complete sequence, cultivar: Nipponbare	Eukaryota, Viridiplantae	NCBI
NC 008401.2	23661561	Oryza sativa Japonica Group DNA, chr. 8, complete sequence, cultivar: Nipponbare	Eukaryota, Viridiplantae	NCBI
NC 008403.2	35571569	Oryza sativa Japonica Group DNA, chr. 10, complete sequence, cultivar: Nipponbare	Eukaryota, Viridiplantae	NCBI
NC 008467.1	35863200	Populus trichocarpa linkage group I, whole genome shotgun sequence	Eukaryota, Viridiplantae	NCBI
NT 024477.14	1034903	Homo sapiens chr. 12 genomic contig, GRCh37.p13 Primary Assembly	Eukaryota, Metazoa	NCBI
NT 024498.12	369930	Homo sapiens chr. 13 genomic contig,	Eukaryota, Metazoa	NCBI
		GRCh37.p13 Primary Assembly		
NT 029928.13	3915179	Homo sapiens chr. 3 genomic contig, GRCh37.p13 Primary Assembly	Eukaryota, Metazoa	NCBI
NT 077528.2	556644	Homo sapiens chr. 7 genomic contig, GRCh37.p13 Primary Assembly	Eukaryota, Metazoa	NCBI
NT 078094.2	868660	Homo sapiens chr. 15 genomic contig, GRCh37.p13 Primary Assembly	Eukaryota, Metazoa	NCBI
NT 167185.1	3353625	Homo sapiens chr. 1 genomic contig, GRCh37.p13 Primary Assembly	Eukaryota, Metazoa	NCBI
NT 167196.1	754004	Homo sapiens chr. × genomic contig, GRCh37.p13 Primary Assembly	Eukaryota, Metazoa	NCBI

We selected 10 popular short-read aligners that employ different algorithmic techniques and indexing structures: SHRiMP2 [1], mrFAST [2], SeqAlto [3], GASSST [4], Bowtie2 [5], BWA-SW [6], SOAP2 [7], CUSHAW2 [8], Masai [9], and Smalt [10].

We used default parameters to run these programs because these aligners appeared to perform well and consistent over the 100 genomes at such settings.

It is not possible to compute the number of *correctly aligned *reads for real reads because positions of real reads in reference genomes are not known. Consequently, precision and recall cannot be computed using real reads. For this reason, we simulated reads for each genome, 2x coverage of reads at lengths 50, 75, and 100 using the *wgsim *program [18]. Reads were generated with default parameters; sequencing error rates equal to 0.5%, 1%, 2%, and mutation rates between 0.1% and 1%, of which 15% are indels. These parameters should be sufficiently realistic for the current sequencing technologies and a large range of organisms.

### Overview performance of aligners

**Figure 1:**
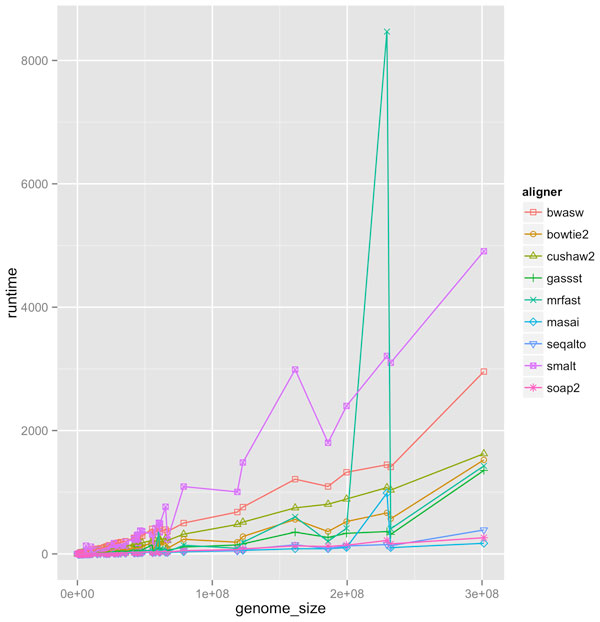
Running time (in seconds) of aligners as function of genome size with 2x coverage, read length equal to 100, sequencing error at 2%, mutation rate at 0.1%.

Figure 1 compares the running times of the aligners as a function of genome size (with 2x coverage). To take advantage of multiple CPU cores, one could manually partition reads into separate sets and run multiple instances across the number of cores. But since some of the aligners were not designed for multiple cores, it made more sense to compare them in single-threaded mode. We found that SHRiMP2 was roughly a magnitude slower than the fastest aligners for larger genomes. Therefore, it was therefore excluded from the figure. Based on running time, Masai, SOAP2 and SeqAlto were among the fastest.

In terms of precision and recall, the average performance over 100 genomic sequences for read lengths 50, 75 and 100 is summarized in Table 4. All aligners were generally very accurate and increasingly so at longer read lengths. On average, CUSHAW2, Masai, and Smalt performed consistently well across read lengths 50-100, whereas Bowtie2, BWA-SW and SeqAlto performed equally well at read lengths 75-100, but were slightly inferior at read length 50. Strictly based on numbers, SHRiMP2 had very good accuracy (in terms of precision and recall), but for larger genomes, it ran very slow. Performance of GASSST seemed peculiar with some recall values larger than 1. This is possible if a read is aligned to multiple locations by the aligner and counted as correct more than once by the SAMtool evaluation package, which allows a gap (default value of 20) between predicted and actual read positions.

**Table 4:** Precision and recall averaged across 100 genomes at read lengths 50, 75, 100.

	Prec-50	Rec-50	Prec-75	Rec-75	Prec-100	Rec-100
Bowtie2	0.9871	0.9062	0.9943	0.9721	0.9965	0.9891
BWA-SW	0.9886	0.8983	0.9952	0.9831	0.9972	0.9951
CUSHAW2	0.9882	0.9868	0.9956	0.9956	0.9975	0.9975
GASSST	0.9836	1.1109	0.9897	1.0339	0.9914	0.9757
Masai	0.9889	0.9861	0.9958	0.9903	0.9976	0.9790
mrFAST	0.9408	0.5700	0.9862	0.9166	0.9833	0.9268
SeqAlto	0.9875	0.8851	0.9956	0.9748	0.9976	0.9925
SHRiMP2	0.9892	0.9798	0.9958	0.9905	0.9975	0.9974
Smalt	0.9858	0.9714	0.9954	0.9944	0.9974	0.9974
SOAP2	0.9893	0.9025	0.9959	0.7904	0.9976	0.6526

In brief, many of these aligners (e.g. Bowtie2, CUSHAW2, SeqAlto) performed similarly accurately on the tested 100 genomes. Without additional information, it can be difficult to decide between these high-performing aligners. It would be useful if we could predict how accurately they perform on new genomes. To explore the aligners' performance on new genomes, we will examine the correlation between various measures of genome complexity and alignment accuracy.

### Correlation between genome complexity and alignment performance

**Figure 2:**
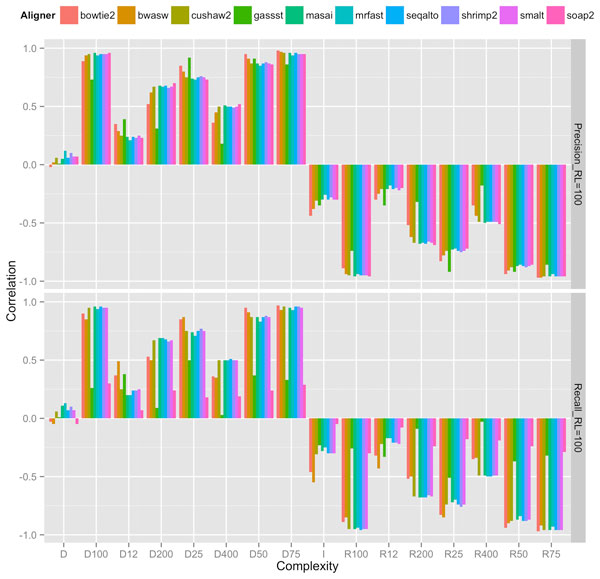
**Correlation coefficients between different measures of complexity and aligners' performance (precision and recall) at read length 100**.

**Figure 3:**
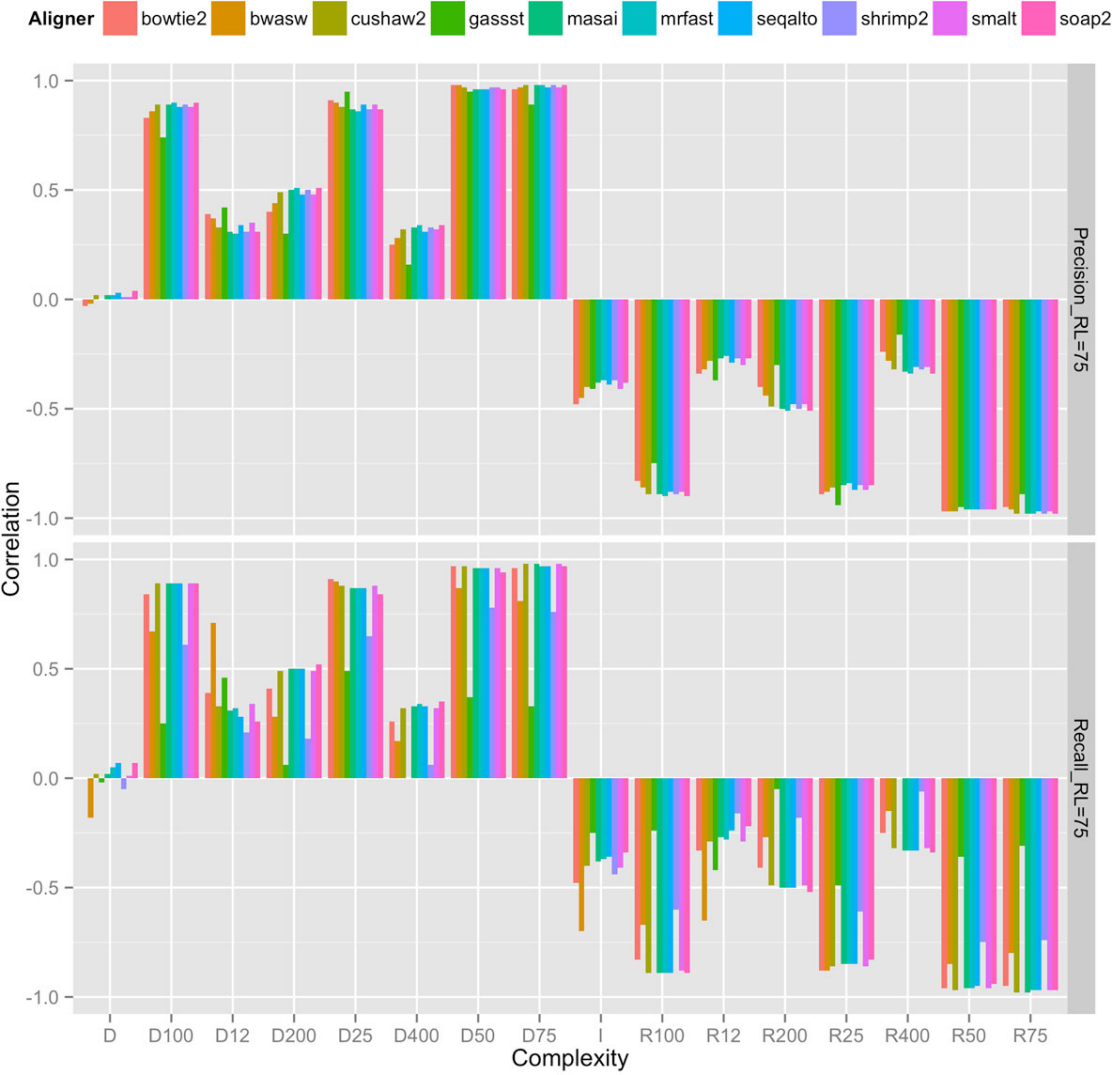
**Correlation coefficients between different measures of complexity and aligners' performance (precision and recall) at read length 75**.

**Figure 4:**
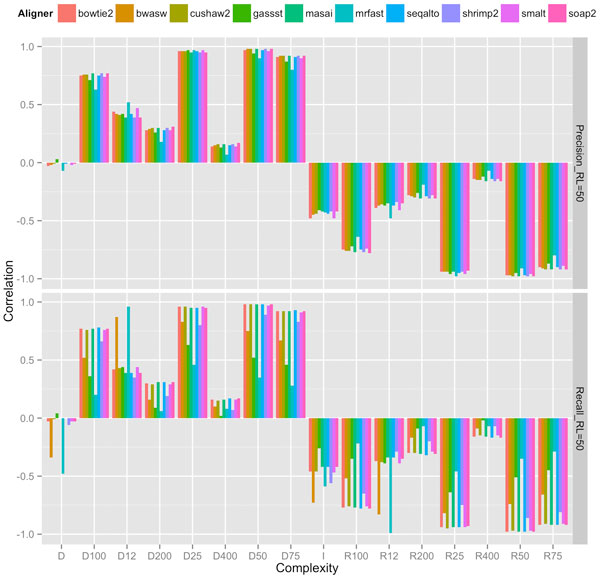
**Correlation coefficients between different measures of complexity and aligners' performance (precision and recall) at read length 50**.

Our experiments showed that an appropriate choice of length-sensitive measure of complexity correlated highly with short-read performance of most aligners across read lengths, rates of mutation and sequencing error. Figures 2, 3, and 4 show the correlation between complexity measures *D_k_, R_k_, D, I *and alignment performance (precision and recall) at read lengths 50, 75, and 100, respectively, for the 100 genomes. We see that the *D*-complexity surprisingly had no correlation to performance across all aligners. The *I*-complexity (Becher et al. [12]) had better but still very low correlation, with correlation coefficients between 0 and -0.3.

We can see that there is a value of *k *for which *D_k _*that correlated highly with performance for both precision and recall, across all read lengths of 50, 75, and 100. For most aligners, the correlation coefficients were approximately 0.95. The only noticeable exception is for GASSST whose correlation coefficients were comparatively lower than those of the others. We think the explanation for this is in GASSST's peculiar performance as we reported earlier, whereby its recalls were above 1 for many of the 100 genomes. Additionally, we could see that when recalls were comparative lower for mrFAST and BWA-SW at read length 50, their correlations were also comparative lower than the other aligners'. It is important to note that some aligners were designed to work optimally with longer reads and consequently do not work very well with shorter reads. One can conclude that if aligners perform predictably in their comfort zones, *D_k _*(or *R_k_*), is a good complexity measure that correlates highly to the accuracy of aligning reads to genomes.

A close examination of the results shows that the value of *k *for which *D_k _*correlated highest with performance was very close to the read length. For example, at read length 100, *D*_100 _had the highest correlations across aligners; at both read lengths 50 and 75, *D*_50 _had the highest correlations, although *D*_25 _also had very high correlations at read length 50. Thus, the most accurate measure of complexity to understand the difficulty of short-read alignment is *length sensitive*. Intuitively, this is because repeats of length *close to *75, for example, influence the accuracy of the alignment of reads of length 75.

The fact that the best value of *k *is less than or equal to read length, and not larger than it implies that *D_k _*accounts for *approximate repeats*. To see this, observe that a 75-mer repeat might not be part of a 100-mer, but surely contains several 50-mer repeats (26 of them, to be precise). This means that *D*_100 _neglects to account for several 50-mers, whereas *D*_50 _accounts for all of these, and these 50-mer repeats directly have an influence of the accuracy of aligning reads of length 75. This is probably why *D*_50 _had a better correlation profile to complexity than *D*_100 _did. The fact that *D_k _*accounts for *approximate *repeats longer than *k *can be explained formally by the so-called *q-gram lemma*, which states that two sequences of length *k *with edit distance *e *share at least *n *- *q *+ 1 - *qe q*-grams. An estimate of complexity involving counting *approximate *repeats might give better correlation. However, the computing of approximate repeats is computational expensive compared to linear time computation of *D_k_*. The best computation of approximate repeats we know of using a lossless filter [19] has an average running time of *O*(*n*^2^). For long genomes, this running time is not desirable. Since *D_k _*and *R_k _*already correlated quite highly (approximately 0.95) for many aligners, a more efficient running time (linear instead of quadratic) seemed to be a better trade-off than a potentially better correlation.

**Figure 5:**
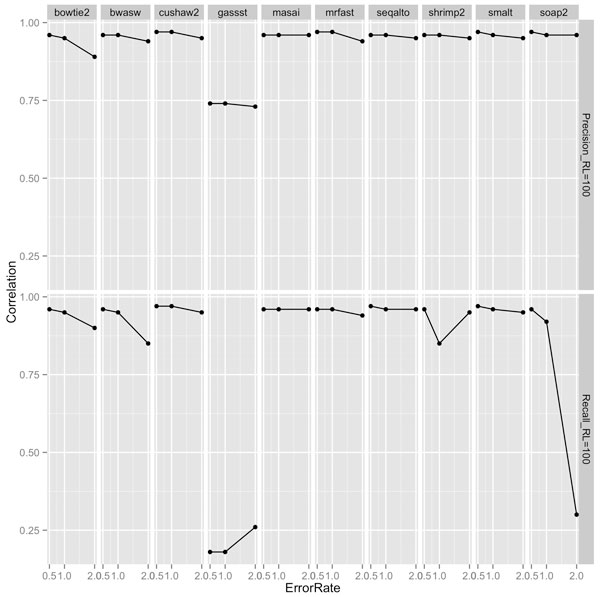
Correlation coefficients between *D*100 and aligners' performance (precision and recall) of aligning reads of length 100 at sequencing error rates of 0.5%, 1%, and 2%.

**Figure 6:**
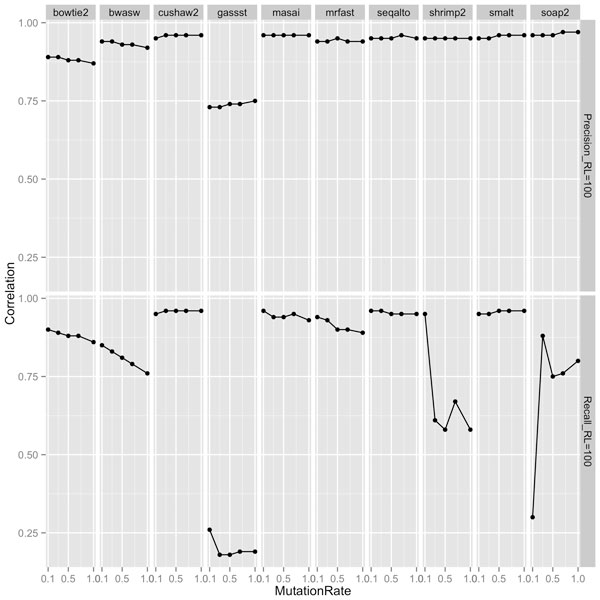
Correlation coefficients between *D*_100 _and aligners' performance (precision and recall) of aligning reads of length 100 at mutation rates between 0.1% and 1%.

At different rates of sequencing error and mutation, respectively, we observed similarly high correlation between performance of the aligners and length-sensitive measures of complexity. To study the correlation at different rates of sequencing error and mutation rates, respectively, we chose to correlate *D*_100 _and alignment performance on reads of length 100. This case is representative for the correlation between the most appropriate length-sensitive measure and aligners' performance at a given read length. Figure 5 and 6 show the correlation between *D*_100 _and aligners' performance of aligning reads of length 100 at different sequencing error rates and different mutation rates, respectively. Across all rates of sequencing error and mutation, the correlation between precision of all aligners and *D*_100 _ranged from high to very high. The lowest correlation was obtained for GASSST at about 0.75. Correlations for the other aligners were around 0.95. Similarly, the correlation between recall and *D*100 was also high for almost all aligners. Overall, compared to precision, recall was, however, not as highly correlated. This might be explained by some aligners' conservative strategies, which aim to make few false positive alignments at the expense of making more false negatives. Further, as expected, at higher rates of sequencing error and mutation, respectively, correlation between performance and complexity decreased. Although, this decrease in correlation is affected more by increasing sequencing errors and by increasing mutations.

### Predicting aligner performance for unknown genomes

The existence of many short-read aligners makes it challenging for researchers to pick the best one for their genomes of interest. Surveys such as [20] compared popular software packages on a few known genomes and served as a good starting point. But when it comes to adopt a particular software package, the decision seems to be a mixture of many factors including the authors' reputation, past familiarity with the software, its alignment accuracy, its quality and ease of use, its resource usage (running time and RAM), and recommendations of fellow researchers. Our focus is on accuracy, defined in terms of precision and recall.

**Figure 7:**
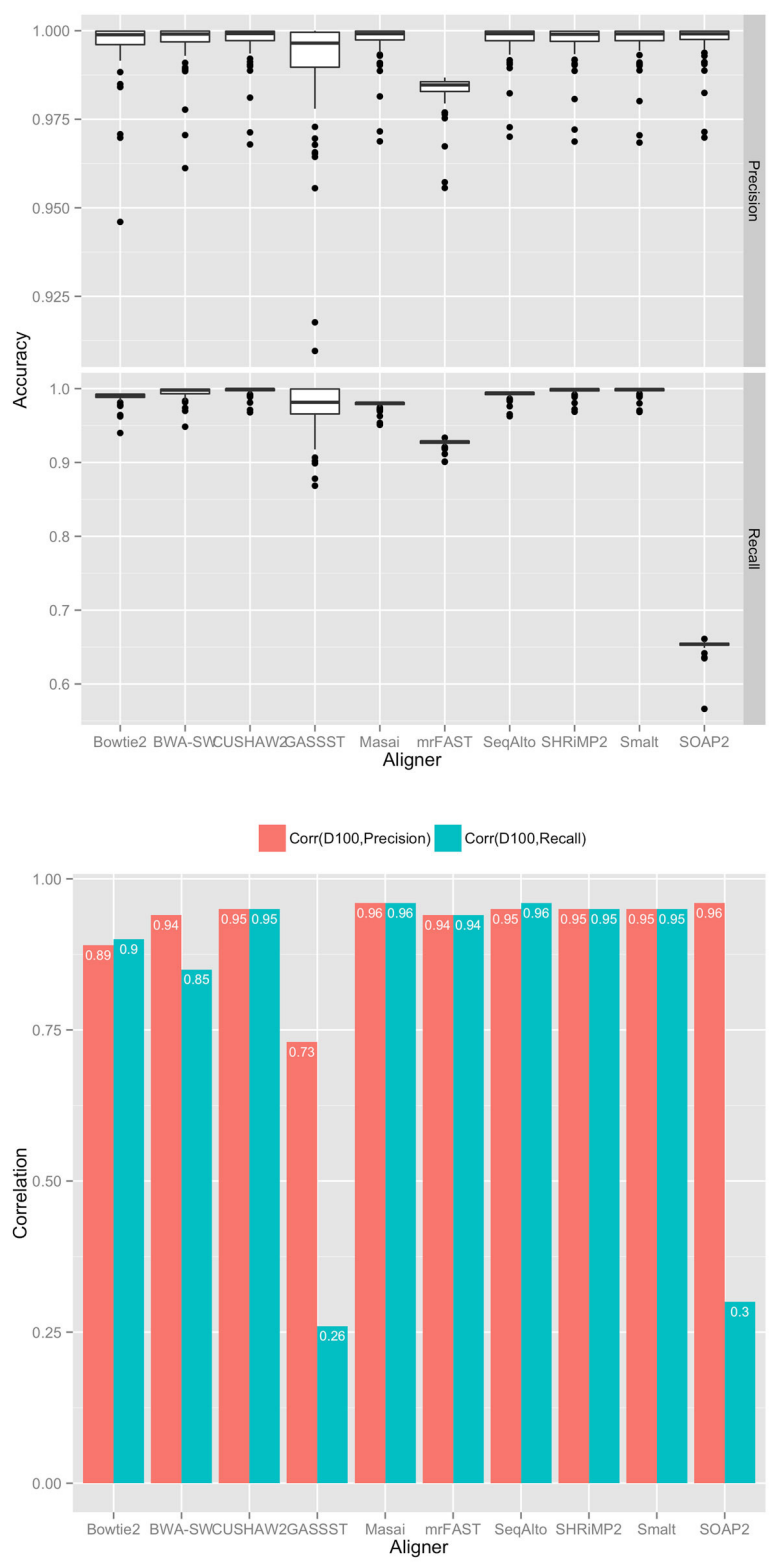
**Top Figure: box plots of accuracy (precision and recall) of aligners across 100 genomes; read length equal to 100**. Bottom Figure: correlation between performance and *D*_100_.

To predict accuracy of a particular aligner on unknown genomes, researchers currently rely on its accuracy on known genomes. Such prediction can be based on summary statistics such as the top figure in Figure 7. This figure shows precisions and recalls of the aligners across 100 genomes in a boxplot figure, which shows medians, interquartile ranges among other statistics. Considering both statistics on precision and recall, we can see that with the exception of MrFast and SOAP2 (and maybe GASSST), the rest of the aligners had similar precisions and recalls across the 100 genomes. While the aligners' performance appeared similar on known genomes, what remains uncertain is, however, how well they perform on *new *genomes.

To remove this uncertainty and make more informed decisions, we might additionally incorporate correlation between genome complexity and accuracy. To illustrate this strategy, consider the bottom figure in Figure 7 shows the correlation between *D*_100 _(since it is the best at read length 100) of the aligners' precision and recall across the 100 genomes. Comparing the performance of the high-performing aligners identified in the previous step (those other than MrFast and SOAP2), we can see that they have different levels of correlations. For instance, Bowtie2 had noticeably lower correlations (0.89 for precision and 0.90 for recall) than CUSHAW2 for both precision and recall). Thus, although Bowtie2 and CUSHAW2 had similar accuracies for the 100 genomes, we expect that CUSHAW2 will more likely have similar accuracies for unknown genomes.

### The effect of *k *on *D_k _*and *R_k_*

**Figure 8:**
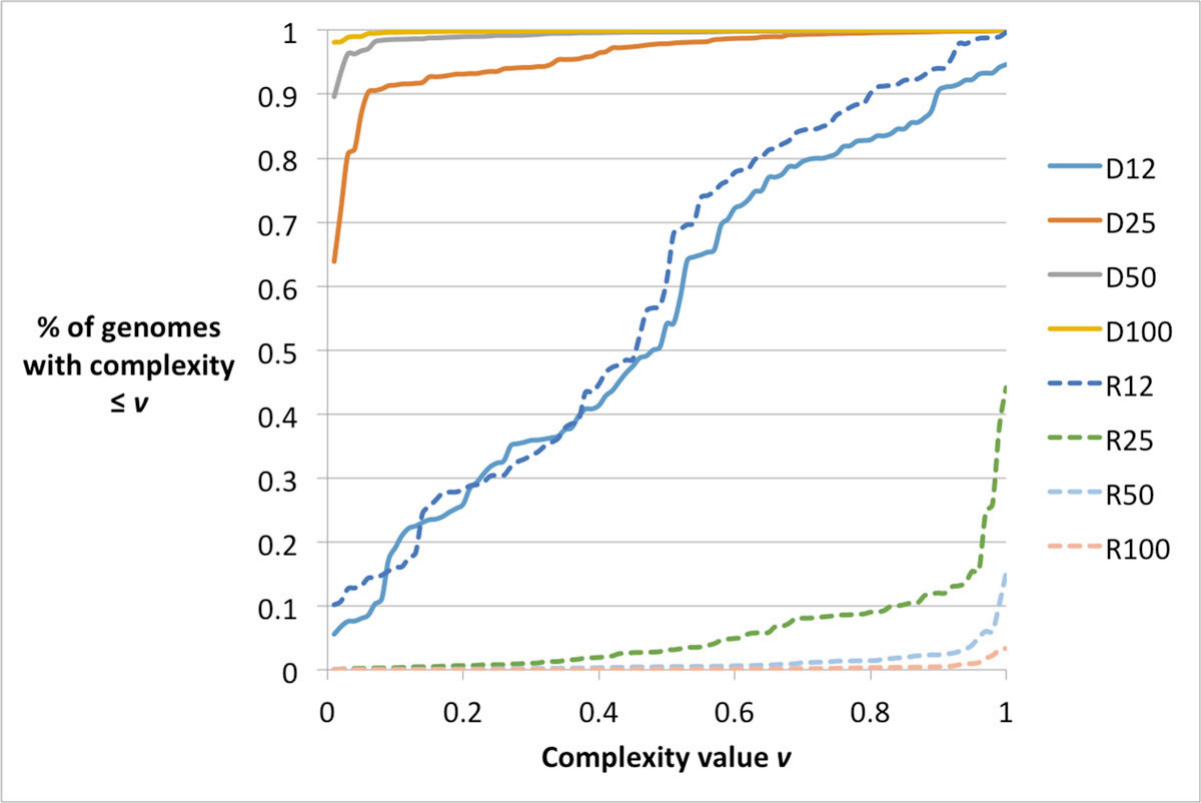
**Cumulative distributions of *D_k _*and *R_k _*(*k *= 12, 25, 50, 100) for 100 genomes**.

Measures *D_k _*and *R_k _*are *length specific *and may have different characteristics for different values of *k*. Figure 8 shows the cumulative distributions of *D_k _*and *R_k _*with *k *= 12, 25, 50, 100. We can see that the distributions of *D_k _*and *R_k _*are similar, but in an *opposite *fashion. For *D*_12 _or *R*_12_, the distribution of complexity of the 100 genomes is quite uniform across the range from 0 to 1. With *k *> 12, however, the distribution is quite non-uniform. As *k *becomes larger, the distribution of *D_k _*(or *R_k_*) becomes much more concentrated toward 1 (or 0).

The transition from near-uniform distributions of *D*_12 _(and *R*_12_) to very skewed distributions of *D_k _*(and *R_k_*), for *k *≥ 50, might explain for the low correlation of *D*_12 _(and *R*_12_) to alignment accuracy. Thus, we can stipulate that right choice of *k *is essential for correlating complexity and alignment accuracy. The right choice of *k *appears to be similar to read length as we have observed.

## Conclusions

We demonstrated that *length sensitive *measures were suitable for studying how genome complexity affected the of short-read alignment. This work has implications for theoretical studies of genome complexity, as well as for comparing alignment methods, and designing cost-effective experiments to assemble genomes. Beyond short-read alignment, these measures should be useful for problems such as short-read assembly, which are affected by genomic repeats.

This method depends on the simulation of reads with known alignment locations, from which we can compute the number of correctly aligned reads for the calculation of precision and recall. With real reads, we cannot know this information. Better simulation of reads will improve the predictive power of this method.

## Competing interests

The authors declare that they have no competing interests.

## Authors' contributions

VP designed methods, experiments, evaluations. SG, NSV, QT selected data, aligners and performed experiments.

## Declarations

Publication charges for this work were funded by NSF grant CCF-1320297 to VP.

This article has been published as part of *BMC Bioinformatics *Volume 16 Supplement 17, 2015: Selected articles from the Fourth IEEE International Conference on Computational Advances in Bio and medical Sciences (ICCABS 2014): Bioinformatics. The full contents of the supplement are available online at http://www.biomedcentral.com/bmcbioinformatics/supplements/16/S17.

## References

[B1] David M Dzamba M Lister D Ilie L Brudno M SHRiMP2: sensitive yet practical short read mapping Bioinformatics 2011 27 7 1011 1012 21278192 10.1093/bioinformatics/btr046

[B2] Alkan C Kidd JM Marques-Bonet T Aksay G Antonacci F Personalized copy number and segmental duplication maps using next-generation sequencing Nat Genet 2009 41 10 1061 1067 19718026 10.1038/ng.437 PMC2875196

[B3] Mu JC Jiang H Kiani A Mohiyuddin M Asadi NB Wong WH Fast and accurate read alignment for resequencing Bioinformatics 2012 28 18 2366 2373 22811546 10.1093/bioinformatics/bts450 PMC3436849

[B4] Rizk G Lavenier D GASSST: global alignment short sequence search tool Bioinformatics 2010 26 20 2534 2540 20739310 10.1093/bioinformatics/btq485 PMC2951093

[B5] Langmead B Salzberg SL Fast gapped-read alignment with bowtie 2 Nat Methods 2012 9 4 357 359 22388286 10.1038/nmeth.1923 PMC3322381

[B6] Li H Durbin R Fast and accurate long-read alignment with burrows-wheeler transform Bioinformatics 2010 26 5 589 595 20080505 10.1093/bioinformatics/btp698 PMC2828108

[B7] Li R Li Y Kristiansen K Wang J SOAP: short oligonucleotide alignment program Bioinformatics 2008 24 5 713 714 18227114 10.1093/bioinformatics/btn025

[B8] Liu Y Schmidt B Long read alignment based on maximal exact match seeds Bioinformatics 2012 28 18 318 324 22962447 10.1093/bioinformatics/bts414 PMC3436841

[B9] Siragusa E Weese D Reinert K Fast and accurate read mapping with approximate seeds and multiple backtracking Nucleic Acids Res 2013 41 7 e78 23358824 10.1093/nar/gkt005 PMC3627565

[B10] Ponstingl H Ning Z SMALT-a new mapper for DNA sequencing reads F1000 Posters 2010 1 313

[B11] Yu X Guda K Willis J Veigl M Wang Z Markowitz MD How do alignment programs perform on sequencing data with varying qualities and from repetitive regions? BioData Min 2012 5 1 6 22709551 10.1186/1756-0381-5-6 PMC3414812

[B12] Becher V Heiber PA A linearly computable measure of string complexity Theoretical Computer Science 2012 438 62 73

[B13] Chor B Horn D Goldman N Levy T Massingham T Genomic DNA k-mer spectra: models and modalities Genome Biology 2009 10 10 R108 19814784 10.1186/gb-2009-10-10-r108 PMC2784323

[B14] Kurtz S Narechania A Stein JC Ware D A new method to compute k-mer frequencies and its application to annotate large repetitive plant genomes BMC Genomics 2008 9 517 18976482 10.1186/1471-2164-9-517 PMC2613927

[B15] Whiteford NE Haslam NJ Weber G Prugel-Bennett A Essex JW Neylon C Visualizing the repeat structure of genomic sequences Complex Systems 2008 17 4 381 398

[B16] Kärkkäinen J Sanders P Burkhardt S Linear work suffix array construction J ACM 2006 53 6 918 936

[B17] Kasai T Lee G Arimura H Arikawa S Park K Linear-time longest-common-prefix computation in suffix arrays and its applications Proceedings of the 12th Annual Symposium on Combinatorial Pattern Matching Lecture Notes in Computer Science 2001 181 192

[B18] Li H Handsaker B Wysoker A Fennell T Ruan J Homer N The sequence alignment/map format and samtools Bioinformatics 2009 25 16 2078 2079 19505943 10.1093/bioinformatics/btp352 PMC2723002

[B19] Peterlongo P Sacomoto GA do Lago AP Pisanti N Sagot MF Lossless filter for multiple repeats with bounded edit distance Algorithms Mol Biol 2009 4 3 19183438 10.1186/1748-7188-4-3 PMC2661881

[B20] Li H Homer N A survey of sequence alignment algorithms for next-generation sequencing Briefings in Bioinformatics 2010 11 5 473 483 20460430 10.1093/bib/bbq015 PMC2943993

